# Prospective study: Impact of breast magnetic resonance imaging on oncoplastic surgery and on indications of mastectomy in patients who were previously candidates to breast conserving surgery

**DOI:** 10.3389/fonc.2023.1154680

**Published:** 2023-03-16

**Authors:** Karina Furlan Anselmi, Cicero Urban, Maíra Teixeira Dória, Linei Augusta Brolini Delle Urban, Ana Paula Sebastião, Flávia Kuroda, Iris Rabinovich, Alessandra Amatuzzi Fornazari Cordeiro, Leonardo Paese Nissen, Eduardo Schunemann, Cleverton Spautz, Julia Di Conti Pelanda, Rubens Silveira de Lima, Mario Rietjens, Marcelo de Paula Loureiro

**Affiliations:** ^1^ Breast Unit, Hospital Nossa Senhora das Graças, Curitiba, Brazil; ^2^ Post-Graduation Program in Biotechnology, Universidade Positivo, Curitiba, Brazil; ^3^ Department of Obstetrics and Gynecology, Universidade Federal do Paraná, Curitiba, Brazil; ^4^ Radiologist of DAPI Clinic, Curitiba, Brazil; ^5^ Department of Pathology, Universidade Federal do Paraná, Curitiba, Brazil; ^6^ Plastic and Reconstructive Surgery Department, European Institute of Oncology, Milan, Italy

**Keywords:** breast magnetic resonance imaging, oncoplastic surgery, preoperative planning, breast conservative surgery, mastectomy

## Abstract

**Background:**

Routine use of magnetic resonance imaging (MRI) in the staging of patients with early breast cancer is still controversial. Oncoplastic surgery (OP) allows for wider resections without compromising the aesthetic results. This study aimed to assess the impact of preoperative MRI on surgical planning and on indications of mastectomy.

**Methods:**

Prospective study including T1-T2 breast cancer patients treated between January 2019 and December 2020 in the Breast Unit of the Hospital Nossa Senhora das Graças in Curitiba, Brazil. All patients had indication for breast conserving surgery (BCS) with OP and did a breast MRI after conventional imaging.

**Results:**

131 patients were selected. Indication for BCS was based on clinical examination and conventional imaging (mammography and ultrasound) findings. After undergoing breast MRI, 110 patients (84.0%) underwent BCS with OP and 21 (16.0%) had their surgical procedure changed to mastectomy. Breast MRI revealed additional findings in 52 of 131 patients (38%). Of these additional findings, 47 (90.4%) were confirmed as invasive carcinoma. Of the 21 patients who underwent mastectomies, the mean tumor size was 2.9 cm (± 1,7cm), with all having additional findings on breast MRI (100% of the mastectomies group vs 28.2% of the OP, p<0.01). Of the 110 patients submitted to OP, the mean tumor size was 1,6cm (± 0,8cm), with only 6 (5.4%) presenting positive margins at the final pathology assessment.

**Conclusion:**

Preoperative breast MRI has an impact on the OP scenario, bringing additional information that may help surgical planning. It allowed selecting the group with additional tumor foci or greater extension to convert to mastectomy, with a consequent low reoperation rate of 5.4% in the BCS group. This is the first study to assess the impact of breast MRI in the preoperative planning of patients undergoing OP for the treatment of breast cancer.

## Introduction

Several prospective and randomized clinical trials have demonstrated equivalence of mastectomy and breast conserving surgery (BCS) in terms of survival and local disease control for the treatment of early breast cancer (1–6). However, up to 20% of patients submitted to BCS would undergo to a second procedure due to the involvement of the margins (7). Thus, accuracy in preoperative local staging is essential for choosing the best surgical treatment for the patient. Although clinical examination, mammography and ultrasound (US) represent the triad traditionally used in the preoperative planning, they fail to assess true tumor size in approximately one-third of patients eligible for BCS (8).

Breast magnetic resonance imaging (MRI) has a high sensitivity (95-100%) in the assessment of tumor extension, multifocality and multicentricity (9–13). However, the routine use of MRI in the staging of patients with early breast cancer is still controversial, as it increases the indications for broader resections and mastectomies (14, 15). Opponents of the routine use of MRI in preoperative staging argue that many of these additional lesions might not have clinical or biological relevance, or even be treated effectively by radiotherapy (16). Regarding the reduction of reoperation rates with the preoperative use of MRI, the literature is also controversial. Some studies failed to show this association (17–19), while others showed a reduction of up to a third of reoperations (20, 21).

Oncoplastic surgery (OP) associates the principles of breast plastic surgery with oncologic surgery and represents an important advance in BCS (22, 23). It allows for wider resections, which results in a lower risk of compromising surgical margins when compared to traditional BCS techniques and improves radiotherapy planning by creating smaller breasts (24–26). However, the accuracy of the imaging methods is also essential in the oncoplastic setting for adequate surgical planning (choosing which pedicles technique and better incision). Studies published to date have not evaluated the use of preoperative MRI in conjunction with OP.

Thus, this study aimed to assess the impact of preoperative MRI on surgical planning and changes in management in patients with early breast cancer and candidates for OP.

## Materials and methods

### Patients

One hundred and thirty-one patients with a diagnosis of T1-T2 breast cancer were enrolled in this prospective study between January 2019 and December 2020 in the Breast Unit of the Hospital Nossa Senhora das Graças in Curitiba, Brazil. All patients had indication for BCS with OP and performed breast MRI before the surgery for the surgical planning. We excluded patients diagnosed with locally advanced or metastatic breast cancer, those who opted for mastectomy despite having an indication for BCS, patients undergoing neoadjuvant chemotherapy, those submitted to breast MRI prior to diagnosis of breast cancer, patients with previous cancer treatment in the breast or other organs, and those with contraindications or limitations for breast MRI (allergy or claustrophobia).

### Breast MRI

Breast MRIs were performed on a 1.5T MRI system (Avanto^®^, Siemens), in a prone position using Ominiscan^®^ contrast (Gadolineo, HE Healthcare) with a dose of 0.2ml/kg and use of an infusion pump with 3ml/s. The imaging protocol included T2-weighted (axial plane) and STIR (sagittal plane) sequences, followed by a dynamic 3D T1-weighted sequence with fat saturation (axial) and immediate reconstruction with subtraction (on pre-contrast sequence and four sequences after contrast, with a time of 90 seconds/acquisition and a total time of 7 minutes). The dynamic sequence was followed by a high-resolution 3D acquisition with fat saturation T1-weighted (sagittal) for reconstruction. Then, all exams were sent to a workstation (Carestrean Health) were the same radiologist, dedicated exclusively to breast imaging, evaluated the morphology and dynamic behavior of the lesions, classifying them according to the BI-RADS^®^ system.

Nonspecific lesions, considered as BI-RADS 3 were not biopsied, following the standard recommendation for this type of lesion. Lesions classified as BI-RADS 4 on MRI were submitted to direct ultrasound (second-look) and percutaneous biopsy. If there was no translation of the lesion on US, they were submitted to percutaneous vacuum biopsy (Mamotomme^®^) guided by MRI or preoperatively marked with technetium and 4% activated charcoal and resected during oncoplastic surgery or mastectomy. Lesions classified as BI-RADS 5 on breast MRI were biopsied preoperatively.

### Pathological analysis

A pathologist with dedication exclusively to breast pathology evaluated the tumor margins in two situations: first, during the intra-operative through imprint and frozen sections and, later, by definitive examination of the paraffin-embedded material. We considered as free-negative margins the tumor distance from the margin above 1mm; narrow when bellow 1mm; positive when carcinoma *in situ* or invasive were detected under the India-painted margin. Additional lesions detected on MRI were evaluated for size and whether it was invasive, *in situ* carcinoma, or a benign lesion.

### Oncoplastic surgical techniques

OP used for conservative surgery were: inferior pedicle, superior pedicle, centralectomy or round-block. Patients undergoing mastectomy were evaluated in terms of tumor size, distance from the tumor to the skin and distance from the tumor to the nipple-areolar complex to decide which type of mastectomy they would be submitted (simple mastectomy, skin-sparing mastectomy or nipple-sparing mastectomy).

### Statistical analysis

Patients were classified according to the following variables: age, menopausal status, family history of breast cancer, body mass index (BMI), breast size (small breasts if bra up to 42, medium if bra between 44 and 46 and large breast if bra 48 or more), type of surgical technique performed, tumor histology, presence of angiolymphatic invasion, margins status and axillary involvement. Family history was considered positive when a first degree relative or two second-degree relatives or male relative with breast cancer were present. The following variables were considered to evaluate the results and compare them with literature: change from BCS to mastectomy; change from unilateral to bilateral mastectomy; wilder resection in conservative surgery (if difference in tumor size between MRI and mammography or ultrasound were greater than 1cm); change in the surgical approach of the contralateral breast; rate of positive margins. We also evaluated the concordance between MRI, mammography, ultrasound and anatomopathological examination in relation to tumor size (variations of up to 5mm were concordant). The analysis of the primary outcome (post-MRI surgical indication) was performed using the Mann-Whitney U test (Wilcoxon Rank Sum Test). Secondary analyzes were designed according to the type of the variable in question: categorical dependent variables were evaluated with Chi-Square Test and Fisher’s Exact Test; continuous variables were evaluated with Mann-Whitney U test (Wilcoxon Rank Sum Test). A *p* value <0.005 was considered significant. The software used was STATA 17.1.

## Results

Between January 2019 and December 2020, we included 131 patients diagnosed with T1-T2 breast cancer and with indication to BCS based on clinical examination and imaging (mammography and ultrasound) findings. After undergoing breast MRI, 110 patients (84.0%) underwent BCS with oncoplastic techniques and 21 (16.0%) had their surgical procedure changed to mastectomy. **Table 1** shows the clinical features of this cohort. The median age was 55.5 years. Most of the patients were postmenopausal (64.9%) and had no family history of breast cancer (74.8%). The mean tumor size on ultrasound, mammography, MRI and anatomopathological examination was, respectively, 1.5cm, 1.4cm, 2.2cm and 1.8cm.

**Table 1:** Clinical and pathological features of study cohort.

Characteristic	No (%)
Median age, years	55.5 ± 10.5
Menopausa status	
Premenopausal	46 (35.1)
Postmenopausal	85 (64.9)
Family history	
Positive	33 (25.2)
Negative	98 (74.8)
Histological subtype	
Invasive ductal carcnome	122 (93.1)
Invasive lobular carcinoma	9 (6.9)
Angiolymphatic invasion	
Present	31 (23.7)
Absent	100 (76.3)
Estrogen receptor	
Positive	118 (90.1)
Negative	13 (9.9)
Progesterone receptor	
Positive	110 (84)
Negative	21 (16)
HER2-neu	
Positive	10 (7.6)
Negative	120 (91.6)
Unkown	1 (0.8)
Tumor size (cm)	
Ultrasound	1.5
Mammography	1.4
Breast MRI	2.2
Pathology	1.8

**Figure 1:**
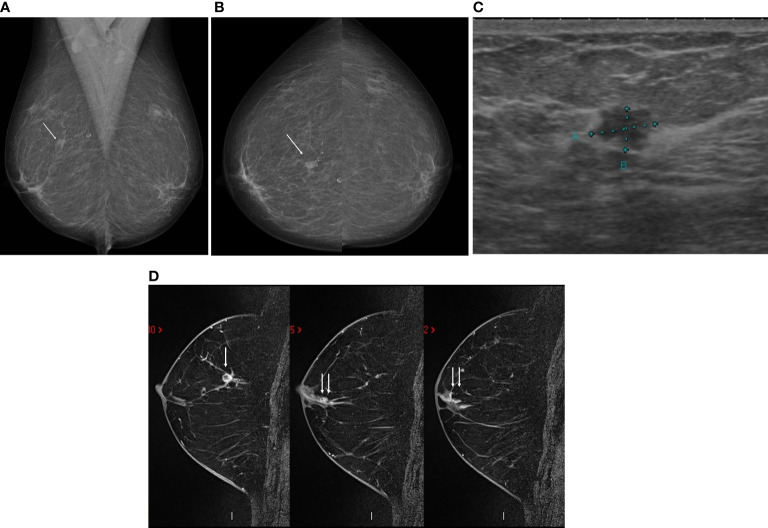
A 58-year-old asymptomatic patient underwent mammography in the mediolateral oblique **(A)** and cranio-caudal **(B)** views, which showed an irregular nodule, low density and indistinct margins in the middle third of the upper quadrants of the right breast (arrow). Ultrasound showed an irregular, hypoechoic nodule, with non-circumscribed margins, parallel to the skin, with no sound change in this topography **(C)**, with percutaneous biopsy demonstrating invasive ductal carcinoma, moderate grade, negative for estrogen and progesterone receptors (Luminal A). Magnetic resonance imaging for staging confirmed the nodule at the junction of the upper quadrants of the right breast (arrow), in addition to demonstrating non-nodular enhancement extending from the nodule to the papilla (double arrow), confirmed as ductal carcinoma *in situ* **(D)**.

Breast MRI revealed additional findings in 52 of 131 patients (38%) (**Figure 1**). The incremental information is shown in **Table 2**. Summarily, breast MRI revealed 29 multifocal lesions (22.1%), 8 multicentric (6.1%), 8 contralateral (6.1%) and 13 patients whose lesions were 1cm larger on MRI when compared with US and mammography (9.9%). Of the 8 patients with multicentric lesions, four also had lesions in the contralateral breast. One patient had multifocal lesions on breast MRI and a contralateral lesion, and another patient had multifocal lesions and a difference in tumor size greater than 1cm on MRI.

**Table 2:** Breast MRI additional findigns in 131 patients.

Type of findings	n (%)
Multifocality	29 (22.1)
Muticentricity	8 (6.1)
Contralateral lesion	8 (6.1)
Altered tumor size	13 (9.9)

Of the 52 additional findings, 47 (90.4%) were confirmed as invasive carcinoma by pathological assessment: 22 of the 29 with multicentric lesions (75.9%); 7 of 8 with multifocal lesions (87.5%); 7 of 8 with contralateral lesions (87.5%) and 11 of 13 with altered tumor size (84.6%). Regarding the contralateral lesions evidenced by MRI, there were 7 cases of invasive carcinoma and 1 case of atypical hyperplasia. Half of the patients underwent mastectomy, and the other half underwent OP.

The surgical procedure was changed to mastectomy in 21 patients (16%): 11 (52.4%) due to multifocality, 7 (33.3%) because of multicentricity, 4 (19%) due to lesions in the contralateral breast and 3 (14.3%) because tumor size was greater than 1cm on MRI in relation to US and mammography. Only in two cases (9.5%), final pathologic analysis did not confirm additional disease seen on MRI. However, these two patients had small breasts and borderline indication for BCS. **Table 3** shows clinical, imaging, and pathological features of patients submitted to OP or to mastectomy. Patients who had their surgical procedure changed to mastectomy had larger tumors on MRI (3.7cm versus 2.0cm, *p*=0.03) and a higher percentage of family history of breast cancer (42.9% versus 21.8%, *p*=0.04). All patients submitted to mastectomy had additional findings on breast MRI, unlike most OP patients (100% vs 28.2%, *p*<0.01). The immunohistochemical subtype did not differ between the types of surgery.

**Table 3:** Clinical, imaging and pathological features of patients submitted to OP or mastectomy.

Characteristic	OP (n=110)	Mastectomy (n=21)	*p*
Median age, y	56.1 ± 10.2	51.7 ± 11.7	0.07
Menopausa status			0.21
Premenopausal	36 (32.7%)	10 (47.6%)	
Postmenopausal	74 (67.3%)	11 (52.4%)	
Family history			0.04
Positive	24 (21.8%)	9 (42.9%)	
Negative	86 (78.2%)	12 (57.1%)	
Histological subtype			0.64
Invasive ductal carcinoma	103 (93.6%)	19 (90.5%)	
Invasive lobular carcinoma	7 (6.4%)	2 (9.5%)	
Tumor size (cm)			
Breast MRI	2.0 ± 1.1	3.7 ± 1.9	<0.01
Pathology	1.6 ± 0.8	2.9 ± 1.7	<0.01
Additional findings on MRI	31 (28.2%)	21 (100%)	<0.01
Multifocality	18 (16.4%)	11 (52.4%)	<0.01
Multicentricity	1 (0.9%)	7 (33.3%)	0.01
Contralateral lesion	4 (3.6%)	4 (19%)	0.02
Altered size	10 (9.1%)	3 (14.3%)	0.44
Positive margins	6 (5.4%)	1 (4.8%)	0.88
Immunohistochemical			
ER positive	101 (91.8%)	17 (80.9%)	0.18
PR positive	93 (84.5%)	17 (80.9%)	0.49
HER2 positive	8 (7.3%)	2 (9.5%)	0.63

OP, oncoplastic; y, years; MRI, magenetic resonance imaging; ER, estrogen receptor; PR, progesterone receptor.

Of the 110 patients submitted to OP, 6 (5.4%) presented positive margins at the final pathology assessment. Of these, two had multifocal tumors on breast MRI, one had a contralateral tumor, and one had a tumor size greater than 1cm on MRI when compared to US and mammography. The remaining two cases did not have additional findings on MRI. One patient submitted to mastectomy had positive margins on final pathology (4.8%). This patient had multifocal lesions on breast MRI and pathological tumor size of 6,0cm.

## Discussion

In this study, preoperative breast MRI did provide relevant additional information for 38% of the patients candidates for BCS with oncoplastic techniques. These additional findings led to a change from BCS to mastectomy in 16% of the cases. The final pathological analysis confirmed invasive carcinoma in 90.5% of the additional findings that led to change in surgical management. This is the first study to assess the impact of breast MRI in the preoperative planning of patients undergoing OP for the treatment of breast cancer.

The studies published to date evaluating the role of preoperative breast MRI have heterogeneous designs and conflicting results, with no consensus on the real role of breast MRI in surgical planning. Regarding the rate of additional findings revealed by breast MRI, our results are in agreement with the POMB Trial, a prospective, randomized, multicenter study, which demonstrated a 38% rate of additional findings (20). The two published metanalysis on this topic separated the rates of multifocal/multicentric lesions from the rates of contralateral lesions (13, 15). The prevalence of additional foci in the same breast ranged from 6% to 34% across studies analyzed by Houssami et al. (median of 16%) and from 6 to 71% across the studies included in Plana et al. metanalysis (mean of 20%). In our study, considering only multifocal or multicentric lesions, the additional detection rate by MRI was 28.2%. Our rate of contralateral lesions (6.1%) was like that found by Plana’s metanalysis (5.5%).

Despite being an important factor, few studies have evaluated the difference in tumor size found by MRI and its impact on changing the surgical management. In our study, 9.9% (13 of 131) of the cases had lesions that were 1cm larger on MRI when compared with US and mammography, a lower rate than that found in the POMB Trial (15%) (20). Several studies draw attention to the tendency of MRI to overestimate tumor size, with overestimation rates ranging from 28% to 33% (27–29). In our study, 11 of the 13 cases (84.6%) had their tumor size confirmed by pathological analysis, indicating a high accuracy of the breast MRI. Furthermore, although 13 patients had their tumor size altered by MRI, this led to a conversion to mastectomy in only 3 cases (2.3% of the total cohort). This may be because all patients underwent OP, which allows wider resections without compromising the aesthetic and functional results of the surgery.

A major concern and discussion in literature is regarding the increase in conversion to mastectomy when breast MRI is routinely used on surgical planning. Our results showed a rate of conversion of 16%, which was similar to that found in the POMB Trial (15%) (20) and slightly higher when compared to two other studies: BREAST-MRI (8.7%) and MIPA Trial (11.3%) (21, 30). Considering wide BCS and contralateral surgeries, in addition to conversion to mastectomy, the preoperative MRI was responsible for changing the surgical approach in 31.1% of patients in BREAST-MRI. MRI correctly modified the surgical procedure in 80% of the cases in this trial. In our study, 90.5% of the additional findings that led to change in surgical management were confirmed by the pathological analysis. The evaluation of MRI images by the same radiologist, with years of experience in breast imaging, may be one of the explanations for the high accuracy of our results.

Interestingly, although our cohort was composed of patients undergoing oncoplastic surgery, this did not reduce the rate of conversion to mastectomy when compared to other studies in the literature. This may be explained by the fact that the decision to perform a mastectomy or BCS is multifactorial. It involves factors besides the imaging findings, such as the patient’s desire, family history of breast cancer, and the relationship between tumor size and breast size.

In our study, patients had a lower reoperation rate due to positive margins (5.4%), when compared to the rates of BCS, whose values vary from 20% to 30% (7, 31, 32). This might be explained by two main factors: the use of OP and the role of MRI in preoperative planning. Several studies have already shown that OP significantly reduces the rates of positive margins and re-excisions, as it allows for larger resections with better aesthetic results (33–35). Our study was not comparative, as all patients underwent preoperative breast MRI. Therefore, we could not measure the impact of MRI in reducing reoperation rates in the scenario of OP. The trials published to date were performed with patients undergoing standard BCS and show conflicting results regarding the role of preoperative MRI in reducing reoperation rates. The POMB Trial, a randomized study, and the MIPA Trial, an observational study with 5896 patients, demonstrated a significant reduction in the reoperation rate in the group undergoing preoperative MRI (5% versus 15% and 8.5% versus 11.7%, respectively) (20, 21). Conversely, other studies have not demonstrated an impact of MRI on reoperation rates (18, 19, 30).

There are some limitations to the present study. First, the study was conducted in a single center, which limits the generalizability of the data. Second, this was a prospective observational study, with no control group. Therefore, the results found here and the real impact of preoperative MRI in the OP setting will require further studies to be confirmed. On the other hand, our study has several strengths, for example, the evaluation of all MRI images by a dedicated exclusively to breast imaging. Another strength is that this is the first study to assess the impact of preoperative MRI in patients undergoing OP. After several studies have already proven that OP are not only oncologically safe, but also improves the aesthetic and functional results, these techniques have been increasingly used and are part of the day-to-day treatment of breast cancer.

In conclusion, our study demonstrated that preoperative breast MRI has a positive impact on the OP scenario, bringing additional information that may help the surgeon in the surgical planning. It allowed selecting the group with additional tumor foci or greater extension to convert to mastectomy, with a consequent low reoperation rate in the BCS group, which is important in the OP scenario.

## Data availability statement

The raw data supporting the conclusions of this article will be made available by the authors, without undue reservation.

## Ethics statement

The studies involving human participants were reviewed and approved by Universidade Positivo. The patients/participants provided their written informed consent to participate in this study. Written informed consent was obtained from the individual(s) for the publication of any potentially identifiable images or data included in this article.

## Author contributions

KA, CU, LU, MR and ML conceived of the original idea. KA, CU, MD, FK, IR, AC, LN, ES, CS and RD constitute the surgical team responsible for all the patients included. LU evaluated all the breast imaging exams. AS evaluated did the pathological analysis of all tumors included. JP and MD helped with the statistical analysis. MD and KA wrote the manuscript with support from CU and LU. RD, MR and ML supervised the project. All authors discussed the results and contributed to the final manuscript. All authors contributed to the article and approved the submitted version.
